# A fluorescent reporter system enables spatiotemporal analysis of host cell modification during herpes simplex virus-1 replication

**DOI:** 10.1074/jbc.RA120.016571

**Published:** 2021-01-07

**Authors:** Katharina M. Scherer, James D. Manton, Timothy K. Soh, Luca Mascheroni, Vivienne Connor, Colin M. Crump, Clemens F. Kaminski

**Affiliations:** 1 Department of Chemical Engineering & Biotechnology, University of Cambridge, Cambridge, UK; 2 MRC Laboratory of Molecular Biology, Cambridge, UK; 3 Department of Pathology, University of Cambridge, Cambridge, UK

**Keywords:** herpesvirus, fluorescence, microscopy, microscopic imaging, host–pathogen interaction, organelle, cytoskeleton, AC, assembly compartment, APS, ammonium persulfate, BAC, bacterial artificial chromosome, BSA, bovine serum albumin, DMEM, Dulbecco's modified eagle's medium, EEA1, early endosome antigen 1, eYFP, enhanced yellow fluorescent protein, FBS, fetal bovine serum, FOV, field of view, gM, glycoprotein M, gC, glycoprotein C, HCMV, human cytomegalovirus, hpi, hours post infection, HFF, human foreskin fibroblast, HSV-1, herpes simplex virus-1, ICP0, infected cell protein, MAVS, mitochondrial antiviral-signaling protein, mIFP, monomeric infrared fluorescent protein, MOI, multiplicity of infection, MTOC, microtubule organizing center, PBS, phosphate buffered saline, PFU, plaque forming unit, RIG-1, retinoic acid-inducible gene I, RLR, RIG-1-like receptors, SIM, structured illumination microscopy, SiR, silicon rhodamine, TEMED, tetramethylethylenediamine, TGN, trans-Golgi network, TGN46, trans-Golgi network protein 46, TOM20, translocase of the outer membrane

## Abstract

Herpesviruses are large and complex viruses that have a long history of coevolution with their host species. One important factor in the virus–host interaction is the alteration of intracellular morphology during viral replication with critical implications for viral assembly. However, the details of this remodeling event are not well understood, in part because insufficient tools are available to deconstruct this highly heterogeneous process. To provide an accurate and reliable method of investigating the spatiotemporal dynamics of virus-induced changes to cellular architecture, we constructed a dual-fluorescent reporter virus that enabled us to classify four distinct stages in the infection cycle of herpes simplex virus-1 at the single cell level. This timestamping method can accurately track the infection cycle across a wide range of multiplicities of infection. We used high-resolution fluorescence microscopy analysis of cellular structures in live and fixed cells in concert with our reporter virus to generate a detailed and chronological overview of the spatial and temporal reorganization during viral replication. The highly orchestrated and striking relocation of many organelles around the compartments of secondary envelopment during transition from early to late gene expression suggests that the reshaping of these compartments is essential for virus assembly. We furthermore find that accumulation of HSV-1 capsids in the cytoplasm is accompanied by fragmentation of the Golgi apparatus with potential impact on the late steps of viral assembly. We anticipate that in the future similar tools can be systematically applied for the systems-level analysis of intracellular morphology during replication of other viruses.

Herpesviruses are large DNA viruses that are typified by their ability to establish both lytic and latent infection cycles. For the prototypical herpesvirus, herpes simplex virus-1 (HSV-1), lytic infections occur in epithelial cells, whereas life-long latent infection is established in sensory neurons. During the lytic infection cycle, the activity of herpesvirus gene products profoundly alters cellular physiology, causing dramatic modifications of the infected cell that convert it into an efficient virus-producing factory. However, there is little understanding of how morphological changes to the host cell relate to key stages and mechanisms of virus replication (1). One of the few examples is the rearrangement of the nucleus and its significance for nuclear capsid egress. Host chromatin is displaced to the nuclear periphery, and the nuclear volume is increased in order to form viral DNA replication compartments during replication (2). These structural changes facilitate transport of newly assembled capsids to the inner nuclear membrane to promote exit from the nucleus (3, 4).

Further key assembly events such as tegumentation and secondary envelopment occur in the cytoplasm (5, 6). As a consequence, almost all host cell organelles and the cytoskeleton are utilized by the virus for transport as well as viral envelope protein synthesis, maturation, and transport or act as major antiviral and inflammatory signaling platforms leading to remodeling of host cell architecture. In order to analyze the correlation between remodeling events and crucial steps in the viral replication cycle, a systematic way of ordering these events with respect to their spatiotemporal occurrence is needed. Because the problem is so complex, the research focus in the past was on usually one, or at most a few, cellular structures. Thus, comparison and correlation of results obtained from previous studies are rendered nearly impossible due to heterogeneous experimental conditions and techniques. Furthermore, it has become apparent over the last few years that intracellular membrane compartments engage in extensive communication, often through direct contact sites (described as the organelle interactome (7)). This concept opens up a new perspective on interorganelle communication, which will influence our understanding of herpesvirus morphogenesis provided an approach to map of organelle changes over the time course of infection is available.

While imaging is the ideal tool to study intracellular cytoskeletal and organelle morphology, there are more than just the technical challenges in capturing cellular rearrangements over the whole viral replication cycle (12 h for HSV-1) systematically and in great detail. Specifically, the large variation between individual cells in a population caused by the asynchronous progression of infection substantially complicates such studies. A measure that is usually used to indicate the state of replication during an experiment is hours post infection (hpi). However, experimental conditions such as the multiplicity of infection (MOI) as well as the cell type used influence the dynamics of replication. To address these issues, two approaches are possible. On the one hand, a multiplexed acquisition with multiple color channels (as shown by Valm *et al.* (7)) can be combined with time-lapse imaging over several hours in order to follow the remodeling of several host cell compartments simultaneously. Alternatively, a universal reference point or temporal marker would allow correlating different imaging sets. The advantage of the second approach is that it also works in fixed cells, which allows higher throughput.

In this study, we have constructed a dual-fluorescent reporter virus tagging an immediate early protein and a true late protein. The distinct temporal and spatial expression patterns of these two fluorescently tagged reporter proteins provide an intrinsic timestamp enabling a simple classification of four clearly separable stages of infection. Applying this classification scheme, we demonstrate the shift in replication kinetics for the different infection stages under various viral loads. By use of structured illumination microscopy (SIM) and light sheet microscopy in combination with expansion microscopy, we generate a detailed and comprehensive map of virus-induced structural rearrangement of the cytoskeleton, secretory pathway compartments, as well as antiviral and inflammatory signaling platforms. Through this chronological sorting, we are able to intercorrelate changes for single organelles and the cytoskeleton allowing us to detect distinctive patterns. We uncover two remodeling phases during which concerted and dramatic relocation of the majority of cellular organelles occurred. During transition from early to late gene expression, early endosomes, mitochondria, and microtubules rearrange around the Golgi compartment. The second change is driven by the fragmentation of the Golgi complex, which leads to a spread of membrane compartments enriched with viral glycoproteins and to a redistribution of the attached organelles. Finally, we analyze by use of a dual-fluorescent virus with fluorescent labels tagged to a capsid protein and a viral glycoprotein when capsids escape the nucleus with respect to the stages of infection as identified by fluorescent timestamping.

## Results

### Timestamping the viral replication state

**Figure 1:**
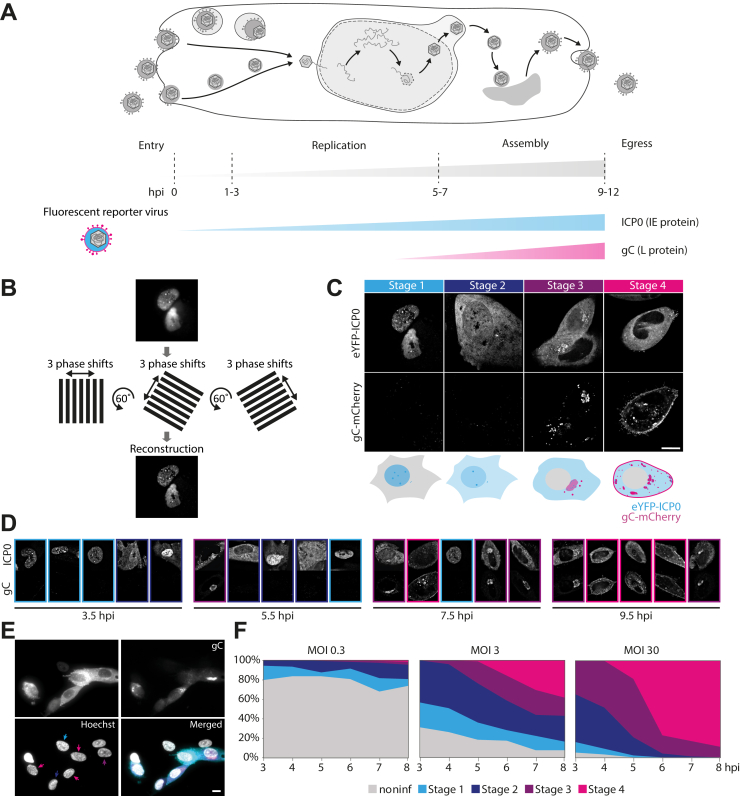
**Timestamping the viral replication state inside individual cells.***A*, schematic illustration of HSV-1 replication mechanism and the engineered reporter virus expression pattern. Fluorescently labeled Immediate early (ICP0) and late (gC) gene expression products mark different stages during lytic infection. *B*, structured illumination microscopy (SIM) was used to generate high-resolution images with low background. *C*, classification using SIM images of HFF cells infected with eYFP-ICP0/gC-mCherry HSV-1. Live cells were imaged at 3.5, 5.5, 7.5, and 9.5 h post infection (hpi). Appearance and localization of the two fluorescently labeled viral proteins can be correlated with four distinct stages in infection (stage 1–stage 4). *D*, representative images taken by SIM (top images show ICP0 and bottom images show gC) and (*E*) widefield microscopy display great variation of infection stages at each time point (hpi). Widefield images were taken at 6 hpi, both SIM and widefield images at MOI 3. Color of arrows indicates the stage of infection. Cells that do not show expression of viral proteins are counted as noninfected. *F*, progression of infection on the single cell level for different multiplicities of infection (MOIs) by classification of widefield images as shown in (*E*). Over 135 cells were analyzed for each time point (3–8 hpi) and MOI (MOI 0.3: n = 255, 245, 297, 240, 204, 264; MOI 3: n = 202, 223, 213, 190, 193, 161; MOI 30: n = 135, 194, 166, 137, 161, 169). Scale bar 10 μm.

For a direct visual readout of the replication state on the single cell level, we developed a fluorescent reporter virus (Fig. 1*A*). As an indicator of early stages of infection, we fluorescently labeled ICP0 with eYFP. ICP0 is one of five immediate early proteins in HSV-1, and it begins to be expressed as soon as the viral genome is deposited inside the nucleus of the host cell. In addition, glycoprotein C (gC) was labeled with mCherry to serve as a marker for the late stages of HSV-1 replication. Single-step growth curve analysis demonstrated nearly identical replication kinetics for the timestamp reporter and wild-type HSV-1 (Fig. S1), suggesting fusion of fluorescent proteins to ICP0, and gC does not impair pathways involved in the replication cycle of HSV-1. We furthermore used time-lapse imaging to confirm the functionality of our construct (Video S1). As expected, at 2 to 3 hpi cells start to exhibit fluorescence from eYFP-ICP0. At a later stage of infection, additional fluorescence signal from gC-mCherry appears. As a virion envelope protein, gC localizes to sites of secondary envelopment.

In order to identify characteristic features in the appearance and redistribution of both proteins with high detail and contrast that appear in a time-dependent pattern during HSV-1 replication, we performed SIM (Fig. 1*B*). We used these visual features to define criteria by which cells could be sorted into four different categories that correspond to distinct stages in the HSV-1 replication cycle. Representative images are shown in (Fig. 1*C*). During stage 1, ICP0 is located almost exclusively in the nucleus and localizes to discrete nuclear structures reminiscent of nuclear domain 10 (ND10) bodies. ICP0 is known to disrupt ND10 body function by causing degradation of components such as PML and Sp100 (8). The second stage in infection is characterized by translocation of ICP0 from the nucleus to the cytoplasm (9). This stage can be visually distinguished from stage 1 by an increase in the cytoplasmic eYFP signal above the background level and simultaneous decrease of the nuclear fluorescence signal. Fig. S2 shows several examples of snapshots taken of cells during stage 2 with varying ratios of nuclear-to-cytoplasmic eYFP-ICP0 intensities. During stage 3, when transition of ICP0 to the cytoplasm is completed, mCherry fluorescence signal appears due to expression of gC. gC is first localized at the juxtanuclear region, where ICP0 often forms several small domains. Usually, cell morphology changes during stage 3, and cells start to round up. Stage 4 is marked by a spread of gC-enriched compartments in the cytoplasm and concentration of gC at the plasma membrane. Cells at that stage are rounded up, show membrane blebbing as well as formation of thin membrane protrusions, and often contain nuclei with indentations.

We detect a high variation between single cell infection stages at each of the distinct times after infection for which we performed imaging. This is consistent for SIM experiments where only single cells can be imaged due to the small field of view (FOV) as well as for widefield microscopy experiments, which allow a higher throughput due to a larger FOV (Fig. 1*D*). We used the new classification scheme and widefield microscopy to characterize the progression of infection on the single cell level for different MOIs. In order to quantify the dynamics of infection progression, we analyzed >135 cells at each time point and MOI (Fig. 1*E*). Not surprisingly, the data show that progression of infection is faster for higher MOIs, which is apparent in the steeper fall of the curves at higher MOIs.

The probability that a cell will become infected can be calculated using the Poisson distribution (10). As the MOI increases, the percentage of cells infected with at least one viral particle also increases and amounts to 26%, 95%, and 100% for MOIs 0.3, 3, and 30, respectively. As Figure 1*E* shows, these predictions are consistent with the experimental findings of our single-cell assay. For all three MOIs, we observe at every hpi of our observation window a combination of individual infection stages. At MOI of 3, we find a relatively balanced mixture of all stages with similar fraction sizes by later stages of the observation time, whereas for the high MOI of 30 late infection stages dominate in the cell population. An intermediate MOI of 3 was used throughout all further experiments to analyze morphological changes over the whole replication cycle.

### Reorganization of the cytoskeleton

**Figure 2:**
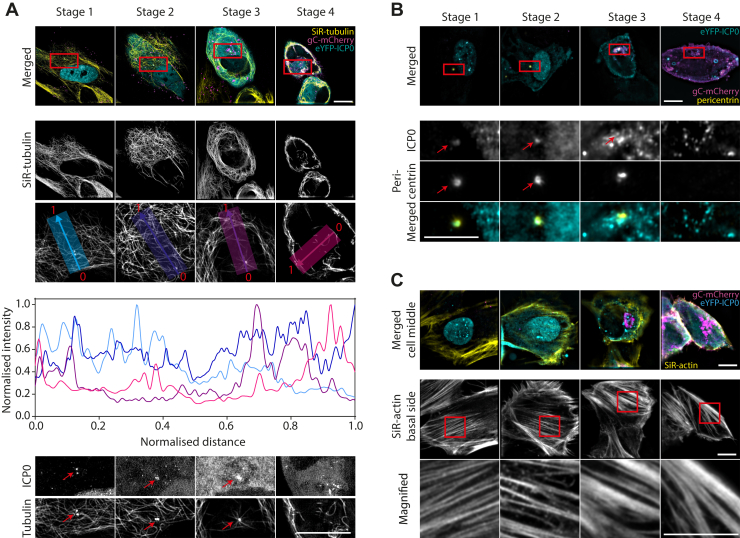
**Reorganization of microtubules, centrosome, and actin stress fibers during infection imaged using a custom-built SIM microscope.***A*, HFF cells were infected with eYFP-ICP0/gC-mCherry HSV-1, stained with SiR-tubulin, and imaged live. At late stages in infection, microtubules are bundled and excluded from the juxtanuclear region as shown by plotting the normalized intensity profiles from the nucleus to the outer cell boundary for each stage of infection. *Bottom row* shows enlarged view of the juxtanuclear region as marked with *red boxes* in the merged images. *Arrows* highlight the colocalization of ICP0 with the microtubule organizing center (MTOC) during the early phase in replication. *B*, HFF cells were infected with eYFP-ICP0/gC-mCherry HSV-1, fixed at 3.5, 5.5, 7.5, and 9.5 hpi, and the centrosome labeled with an antibody for pericentrin (indicated by *red boxes* in the merged images). During stage 1 and 2, colocalization of ICP0 with the centrosome can be detected. *C*, HFF cells were infected with eYFP-ICP0/gC-mCherry HSV-1, stained with SiR-actin, and imaged live. For classification purposes, three-color images of ICP0, gC, and SiR-actin were taken at a middle section of each cell. Shown are the merged images of all three channels (left). Actin stress fibers are remodeled at the basal side of respective cells. *Bottom row* shows enlarged view of actin stress fibers (areas marked with *red boxes* in *upper row*). Scale bars 10 μm.

**Figure 3:**
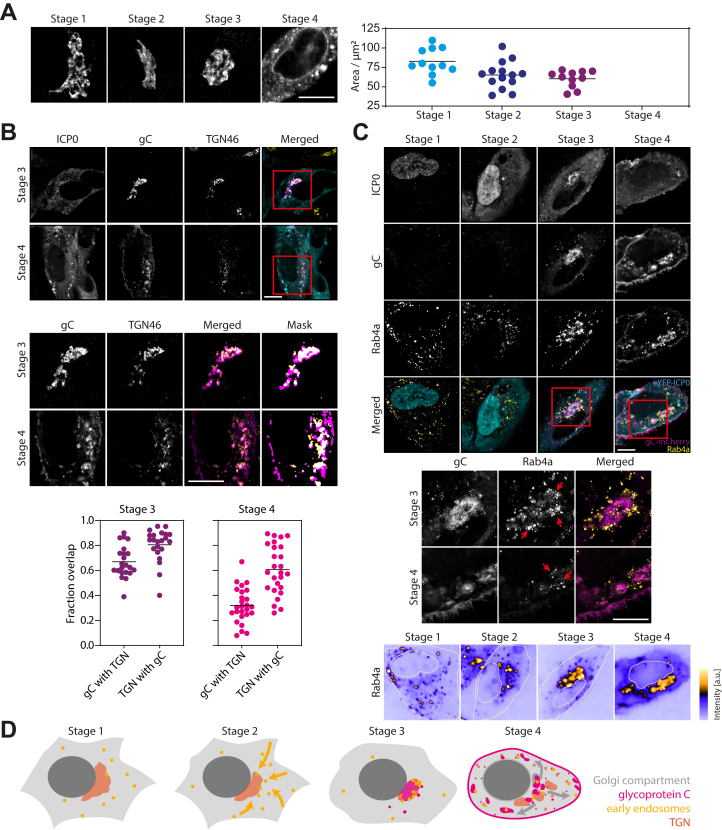
**Remodeling of membrane compartments involved in glycoprotein trafficking and HSV-1 assembly imaged with SIM in fixed cells.***A*, HFF cells were infected with eYFP-ICP0/gC-mCherry HSV-1 and fixed at 3, 6, and 9 hpi. The Golgi apparatus was stained with an anti-58K Golgi protein antibody following standard immunofluorescence protocol and imaged using a custom-built SIM microscope. The 58K Golgi protein marker was used to measure the size of the Golgi compartment over the time course of the replication cycle. The area occupied by the Golgi apparatus was calculated for at least ten cells for each stage (stage 1: n = 11, stage 2: n = 14, stage 3: n = 11). The area continuously decreases from stage 1 to stage 3. During stage 4, the Golgi apparatus was fragmented in all imaged cells (n = 12) and no area was calculated. *B*, object-based colocalization of gC and TGN compartments from SIM data was analyzed by calculating their overlap. Overlap during stage 3 is very high but decreases during stage 4 since gC is trafficked to other cellular membrane compartments such as the plasma membrane. (Stage 3: n= 22 cells, stage 4: n = 26 cells). *C*, accumulation of early endosomes at the juxtanuclear region and spread correlated to fragmentation of juxtanuclear compartment. At stage 1, early endosomes are widely spread in the cytoplasm but start to accumulate at the juxtanuclear region during stage 2. At stage 3, early endosomes are further concentrated at the gC-enriched juxtanuclear region, and Rab4a is found within the assembly compartment. Fragmentation of the assembly compartment leads to respread of early endosomes in the cytoplasm during stage 4. Red arrows in the enlarged view highlight early endosomes clustered around the gC-enriched membrane compartments. The bottom panel illustrates Rab4 density in the cytoplasm, which is homogeneous at stage 1 but increases at the juxtanuclear region and gC-rich compartments during later stages. *D*, schematic illustration of early endosomes, gC, Golgi, and TGN compartments during HSV-1 replication. Scale bars 10 μm.

The cytoskeleton plays an important role during the replication of HSV-1. For effective viral assembly and egress, which take place during the late phases of replication, HSV-1 uses microtubules to transport nucleocapsids to the sites of secondary envelopment. However, it is also known that the cytoskeletal architecture is extensively remodeled during HSV-1 replication (11, 12). By use of our “timestamp” reporter virus, we investigated when and in which way microtubule architecture changed during replication. For imaging, microtubules were stained with SiR-tubulin in cells infected with the reporter virus. SiR-tubulin is a marker for all types of tubulin including microtubule filaments and the centrosome/microtubule organizing center (MTOC, containing gamma-tubulin). Figure 2*A* shows the remodeling of the microtubule network in representative images for the different stages of infection. At the second stage, microtubules start to disconnect from the MTOC. During transition from stage 2 to stage 3, microtubules are cleared from the juxtanuclear region and start to form thick bundles around the juxtanuclear region (see normalized intensity profiles in Fig. 2*A*). Furthermore, SiR-tubulin staining of the MTOC can no longer be observed at stage 4. Interestingly, we observed that ICP0 colocalizes with the MTOC during the early replication stages (Fig. 2*A*). The direct recruitment of ICP0 to this organelle may explain the observed loss of the MTOC and the published role of ICP0 in modifying the microtubule network in HSV-1 infected cells (13). To specifically label the centrosome, cells were immunostained for pericentrin (Fig. 2*B*). This organelle can clearly be observed during stages 1 to 3. However, it disappears during stage 4, similar to SiR-tubulin labeling of the MTOC. Consistent with Figure 3*A* we find that ICP0 colocalizes with pericentrin during early stages in infection.

Actin is an important structural component of the cell and also important for intracellular transport. Since several studies have reported that herpesviruses exploit actin and actin-associated myosin motors for viral entry, intranuclear transport of capsids and egress, we were interested in any morphological changes that HSV-1 induces to the actin cortex and the time at which they occur during replication. We first imaged the middle section of infected cells stained with SiR-actin to classify the stage of infection (Fig. 2*C*). By means of the actin stain, the cell outline is clearly identifiable and shows that cells are rounded up during late stages in infection (stage 3 and 4). This morphological change is reflected in the reorganization of the stress fibers in the actin cortex at the cell base. We observe a transition from thin stress fibers early in infection (stage 1) to thick fibers (stage 4) (Fig. 2*D*). The morphological changes seem to be initiated during stage 2 where many of the stress fibers become branched.

### Transport pathways and potential envelopment compartments

Next, we studied the infection-induced reorganization of compartments involved in glycoprotein trafficking and virus assembly. The assembly of herpesviruses is known to involve the wrapping/budding of nucleocapsids, together with the complex layer of tegument proteins, at membranes derived from post-Golgi endocytic compartments (see reviews by Johnson and Baines (14) and Owen *et al.* (6)). Markers of the Golgi apparatus, trans-Golgi network (TGN), and early endosomes were labeled by immunostaining (58K protein for the Golgi, TGN46 for the TGN, and EEA1 for early endosomes) or tagged with the autofluorescent protein mIFP, which is compatible with the eYFP and mCherry tags of ICP0 and gC (B4GAL-T1 for the Golgi apparatus and Rab4a for early endosomes).

The Golgi apparatus, which is located next to the nucleus and centrosome, is a key component of the secretory pathway. In HSV-1 infection, viral glycoproteins accumulate in the Golgi apparatus where they are modified before transport to the plasma membrane and endosomes. Immunostaining of the 58K Golgi protein yielded a strong fluorescence signal, which allowed the clear identification of the Golgi apparatus (Fig. 3*A*). The mIFP-tagged B4GAL-T1 is concentrated at the juxtanuclear Golgi compartment but is also found in vesicular structures throughout the cytoplasm (Fig. S3*A*). The most apparent change in Golgi morphology takes place during very late stages in infection. At stage 4, the Golgi apparatus fragments into several smaller compartments and a great number of small structures, an event we observed with both Golgi markers (Fig. 3*A* and Fig. S3*A*). The dispersion of the Golgi complex influences the distribution of gC. During stage 3, gC is mainly located at the Golgi compartment. The fragmentation of the Golgi apparatus leads to a spread of the gC-enriched membrane compartments in the cytoplasm of the host cell. Interestingly, by measuring the area occupied by the Golgi apparatus prior to fragmentation at stage 4, we observed a continuous compaction of the compartment from stage 1 to stage 3 (Fig. 3*A*). A change in shape can also be observed with the mIFP-tagged marker B4GAL-T1, where the Golgi complex appears to be more compact across stages 2 to 3. The observed Golgi fragmentation is accompanied by a loss of the centrosome/MTOC (Fig. 2, *A*–*B*).

**Figure 4:**
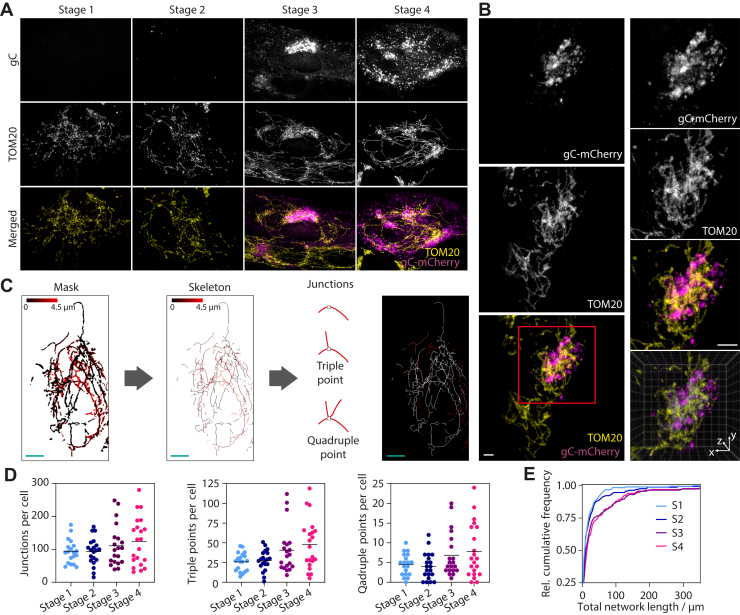
**Interlacing of mitochondria and assembly compartments and spatial distribution of peroxisomes.***A*, HFF cells were infected with eYFP-ICP0/gC-mCherry HSV-1 and fixed at 3.5, 5.5, 7.5, and 9.5 hpi. Mitochondria were labeled by staining TOM20 following standard immunofluorescence protocol, mounted and z-stacks were taken at a widefield microscope. Displayed are maximum intensity projections after deconvolution showing exemplary cells for each stage of infection. From the *top* to *bottom row*: gC, mitochondria (TOM20) and the two channels merged (*magenta*: gC, *yellow*: mitochondria). *B*, infected cells were fixed 9 hpi and immunostained for TOM20 to label mitochondria. mCherry signal was enhanced by use of a nanobooster. Samples were then expanded using a published expansion microscopy protocol (42), and cells in stage 3 imaged using light sheet microscopy (Fig. S5). 3D structure of intertwined mitochondria and sites of secondary envelopment can be clearly observed. *Upper row*, whole cell, *lower row*, juxtanuclear region. *C*, for quantitative analysis of mitochondria networks, first masks were created from z-stacks as described in (*A*), which were then skeletonised. The lookup table (LUT) indicates the height of the mitochondria in z. The skeletons were analyzed to yield information about junctions and networks (LUT indicates individual mitochondria networks within the cell). *D*, scatter plots with indicated mean values show a slight increase in the number of junctions per cell for stages 3 and 4 compared with stages 1 and 2. A stronger increase is noticeable for triple points and quadruple points. *E*, as can be seen from plotting the relative cumulative frequency of the total network length, mitochondria networks increase in total length for stages 3 and 4 compared with stages 1 and 2. (Stage 1: n = 19 cells, stage 2: n = 20 cells, stage 3: n= 20 cells, stage 4: n = 21 cells for (*D*) and (*E*)). Scale bars 10 μm.

The membranes of the TGN are potential compartments for secondary envelopment. We labeled them through immunostaining for TGN46, an established TGN marker, in cells that were fixed 3.5, 5.5, 7.5, and 9.5 hpi and imaged using SIM. The object-based colocalization between TGN46 and gC was measured for infection stages 3 and 4. In a first step, images were segmented to identify TGN and gC compartments, and in a second step, the overlap between those two compartments was calculated. Representative merged images of gC (magenta) and TGN46 (yellow) after segmentation are shown in Figure 4*B* where the overlap is indicated by white pixels. During stage 3, TGN and gC colocalize strongly, whereas overlap of gC compartments with the TGN is a bit lower (mean = 67%) than the converse (mean = 81%). This indicates that TGN compartments are almost completely occupied by the viral envelope protein gC, whereas gC also seems to localize to compartments other than, but close to the TGN. At stage 4, the overlap between gC and TGN (32% for gC with TGN and 61% for TGN with gC, mean values) compartments decreases. This can be explained by trafficking of gC from the TGN to other compartments *e.g.*, the plasma membrane as can be seen in the respective representative image (Fig. 3*B*).

As other potential sources of membrane for secondary envelopment (15, 16), endosomes likely play a key role in HSV-1 assembly. To monitor the spatial distribution of early endosomes during the HSV-1 replication cycle, we generated a stable cell line expressing Rab4a tagged with the autofluorescent protein mIFP. Figure 3*C* shows that Rab4a localization changes dramatically over the viral replication cycle. Figure 3*D* illustrates the Rab4a density distribution for all four infection stages. Rab4a vesicles are of small size and characterized by a strong fluorescence signal. They relocate to the juxtanuclear region during an early stage in infection (stage 2). During stage 3, Rab4a vesicles cluster at and around the gC-enriched assembly compartment at the juxtanuclear region. Very late in infection at stage 4, Rab4a vesicles are redistributed within the cytoplasm due to fragmentation of the gC-enriched TGN compartments around which Rab4 vesicles are still clustered. Furthermore, we detect subregions of weaker Rab4a fluorescence signals within compartments that are likely to become or are enriched with gC (see overlap of gC (magenta) and Rab4 (yellow) within merged images at stages 3 and 4 in Fig. 3*C*). These observations indicate that Rab4 vesicles cluster at gC-rich compartments (red arrows in Fig. 3*C*) and that Rab4 is taken up by those compartments that contain the viral glycoprotein gC. A very similar behavior at all stages was also observed for a second early endosome marker, EEA1, which was monitored in fixed cells using immunostaining (Fig. S3*B*). Taken together, these observations suggest that early endosomes partially fuse with other membranes, presumably derived from the TGN, near the centrosome, perhaps indicating formation of secondary envelopment compartments. Figure 4*E* illustrates the complicated relocation events of early endosome and TGN compartments.

We also studied the location of lysosomes during infection. Lysosomes are trafficked between the juxtanuclear region and cell periphery *via* microtubule-driven transport, and their position is influenced, for example, by nutrient starvation. Imaging of lysosomes stained with SiR-lysosome in live cells revealed a similar behavior to early endosomes (Fig. S3*C*). Lysosomes are directed toward the juxtanuclear region during the early stages of infection and accumulate near gC-rich membrane compartments late in infection. This suggests concentration of all endocytic compartments at the juxtanuclear assembly compartment at stage 3, followed by dispersal during stage 4.

### Antiviral and inflammatory signaling platforms

A link between mitochondrial dynamics and viral infections has been reported for a wide range of viruses (see review by Khan *et al.* (17)). Mitochondria also act as platforms for antiviral immunity through the retinoic-acid-inducible gene I (RIG-I)-like receptors (RLR) and mitochondrial antiviral-signaling protein (MAVS) signal transduction pathway (18). To observe changes in mitochondrial morphology due to infection, cells were stained with MitoTracker Deep Red and imaged using SIM (Fig. S4). We observed infection-induced changes to the mitochondrial network, in particular clustering around the gC-enriched juxtanuclear region. However, 2D imaging using SIM did not allow us to have a full overview of the mitochondrial network. Therefore, in order to gain a better picture of the mitochondrial network topography, we changed the imaging protocol and took z-stacks of infected cells that were fixed and immunostained for the mitochondria marker TOM20 using a widefield microscope. Stacks were then deconvolved and maximum intensity projected (Fig. 4*A*). We observed a strong reshaping of the mitochondria network morphology from a more heterogeneous population of individual organelles (stage 1) to networks (stage 4). In particular, during stage 3, mitochondria appear to be densely concentrated around the gC-enriched juxtanuclear region. Stage 4 showed that these dense mitochondrial networks are pulled apart due to Golgi fragmentation but remain associated with fragmented gC-rich membranes while these dense regions are connected by long tubular mitochondria.

However, the high concentration of mitochondria and gC-positive membranes prevented us from obtaining a clear overview of these convoluted structures using widefield microscopy. To address this, we employed the recently developed technique of expansion microscopy in combination with light sheet microscopy to resolve the 3D arrangement of mitochondria and gC-rich juxtanuclear compartment with much higher resolution. Expansion microscopy (19) involves physical, isotropic swelling of fixed samples that can provide up to 64- to 80-fold increased sample volume, effectively providing super resolution imaging data using conventional fluorescence microscopes (Fig. S5). Using this technique, we demonstrate that mitochondria and viral assembly compartments are intimately intertwined, with the mitochondria interlacing the gC-enriched membranes (Fig. 4*B*). Quantitative analysis of the mitochondria skeletons using deconvolved widefield data (Fig. 4*C*) reveals that the number of junctions per cell, especially the triple and quadruple points, increase during later stages (stage 3 and 4) compared with early stages in infection (Fig. 4*D*). Furthermore, the size of the mitochondria networks is larger for late stages than early stages (Fig. 4*E*). These data show that mitochondria become more interconnected and grow into larger networks centered on gC-enriched membrane compartments during the late stages in infection.

Peroxisomes have also been reported to be important signaling platforms for antiviral innate immunity. We labeled peroxisomes in infected cells by immunostaining for catalase (Fig. S6*A*) and analyzed the dependence of peroxisome size on the stage of infection. However, no obvious change in peroxisome location was observed, except potentially during stage 4 where peroxisomes seem to be located around gC-enriched compartments (Fig. S6*B*). However, this seeming clustering could also be due to infection-induced cell rounding. In addition, we did not observe any significant change in the size of peroxisomes across all four stages of infection, with a consistent organelle median area of 0.20 to 0.22 μm (Fig. S6*C*).

### Correlation of Golgi fragmentation and nuclear egress

**Figure 5:**
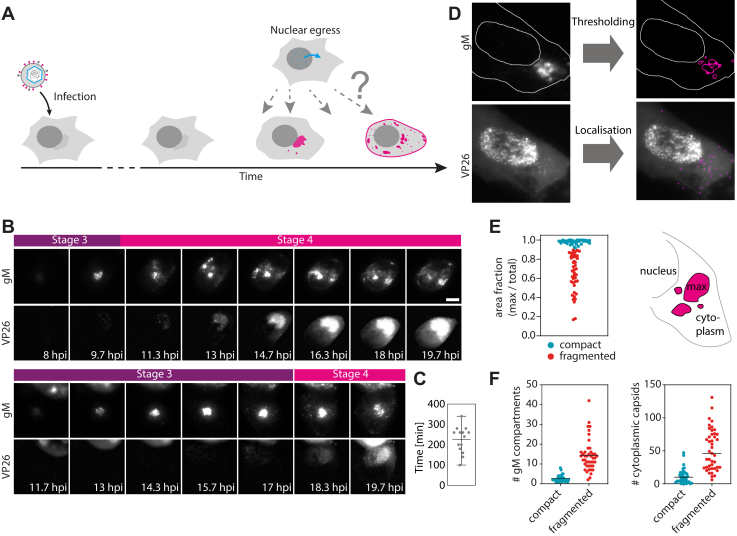
**Temporal correlation between Golgi fragmentation and nuclear egress.***A*, schematic illustration of the problem of putting assembly events into temporal context of general host cell modification processes. *B*, widefield time-lapse imaging of HFF cells that were infected with VP26-eYFP/gM-mCherry HSV-1 at MOI 3. Time lapse was started at 8 hpi and carried out at 37 °C and 5% CO_2_. Images were acquired every 20 min. Scale bar 10 μm. *C*, time between start of gM accumulation at juxtanuclear compartments and fragmentation of those compartments (n = 14 cells). *D*, compartment size was measured by intensity thresholding of gM fluorescence images. Number of cytoplasmic capsids was detected using the Fiji plugin TrackMate. *E*, the area fraction of the maximum sized gM fragment was used to distinguish cells with compact or fragmented Golgi compartments (n = 82 cells). *F*, number of gM fragments as well as cytosolic capsids is largely increased in cells with fragmented compartments. Scale bars 10 μm.

HSV-1 assembly is a complicated and finely orchestrated process. However, regarding spatiotemporal occurrence of the cytoplasmic assembly events, such as tegumentation and secondary envelopment, little is known. These processes are initiated by the nuclear egress of capsids that are formed in the host cell nucleus. Here, we use a dually fluorescently labeled virus to determine how nuclear egress fits temporally into the processes of cytoskeleton and organelle reorganization (Fig. 5*A*). We tagged the capsid protein VP26 with eYFP and the viral envelope protein gM with mCherry. To make sure that fluorescent tagging did not substantially alter replication kinetics compared to wild-type virus, we performed single-step growth curve analysis. This experiment proved very similar replication kinetics for VP26-eYFP/gM-mCherry and wild-type HSV-1 (Fig. S1*B*). Using multiplexed quantitative proteomic analysis over the whole time course of HSV-1 infection, it has recently been shown that the viral glycoproteins gM and gC follow similar kinetic expression profiles (20). We demonstrate first by using time-lapse imaging that indeed gM behaves in a similar way to gC, which allows for spatial and temporal correlation with our previous data sets acquired with the timestamp virus (Fig. 5*B*). gM first accumulates at the juxtanuclear region and fragments later into smaller compartments, which are distributed throughout the host cell cytoplasm corresponding to stages 3 and 4. The time-lapse series also shows that capsids are formed in the nucleus after accumulation of gM at the juxtanuclear region. For individual cells, the time of gM accumulation before fragmentation varies largely and ranges from 90 min to 5.5 h with a mean duration of 3.5 h (Fig. 5*C*). For quantification of gM compartment size and number, fluorescence images were intensity thresholded, and the resulting outline was analyzed using the particle analyzer of Fiji (Fig. 5*D*). In order to determine the number of cytoplasmic capsids, we used the Fiji plugin TrackMate (21). Cells that were infected and fixed at 8.5 hpi were imaged at a widefield microscope and sorted according to fragmentation of the gM compartment (Fig. 5*E*). If the area fraction of the largest gM fragment was 90% or more, the compartment was classified as compact, otherwise it was considered as fragmented. As expected, the number of gM compartments per cell is much higher for cells that are fragmented than cell with compact Golgi compartment. By plotting the number of cytoplasmic capsids in relation to the state of juxtanuclear compartment (Fig. 5*F*), we demonstrate a temporal correlation between nuclear egress and Golgi fragmentation. The results indicate that the nuclear egress of capsids starts during stage 3, where a considerable (average 11 capsids per cell) number of capsids can be found in the cell cytoplasm. This number however is dramatically increased in cells with fragmented Golgi compartment (corresponding to stage 4) with an average number of 51 capsids per cell.

## Discussion

To understand the impact of viruses on host cell biology, as well as the mechanisms behind cytopathic effects and the wider pathogenesis of viral infection, it is vital to accurately define virus-dependent changes to the subcellular architecture of cells. Herpesviruses are large and complex viruses that cause dramatic effects on cell morphology during their lytic replication cycles. However, there is a lack of understanding of when and how the architecture of a cell is changed during the course of herpesvirus infection, and in particular, how specific effects on morphology relate to phases of the replication cycle. We set out to address this by developing a reporter virus that provides an accurate readout of infection stage and advanced high-resolution microscopy analysis of multiple aspects of host cell morphology.

By engineering a virus with fluorescent tags fused to one of the earliest (ICP0) and one of the latest (gC) expressed viral proteins, we have created an intrinsic timestamp reporter of HSV-1 replication, which allows for the accurate determination of the stage in viral replication at the single cell level. Observing the temporal and spatial expression patterns by use of high-resolution fluorescence imaging provides unambiguous visual descriptors to introduce a classification scheme, sorting each cell into one of four distinct stages of infection (stage 1–stage 4). In the future, we envisage opportunities to further refine the classification of HSV-1 replication by including additional fluorescently tagged viral proteins with distinct spatial and kinetic expression profiles as well as through the use of machine learning to automate data categorization (for reviews, see Moen *et al.* (22) and von Chamier *et al.* (23)).

The benefits of our timestamping and classification method were demonstrated by studying the dynamics of viral replication at different MOIs. Our measured data fit well with the theoretically expected infection ratios in the cell population and importantly highlight the degree of heterogeneity between individual cells within infected cell populations with co-occurrence of the different stages of infection at each time point. Without an intrinsic method to categorize the infection stage within each cell, such as our timestamp reporter virus method, attempting to relate any observed cellular changes to viral replication kinetics is fraught with difficulty. While using a high MOI such as 30 PFU/cell provides a seemingly more synchronous infection profile, a substantial increase in the fraction of cells in stage 4 at relatively early times post infection is the main effect, which reduces the opportunity to observe early stages of the infection process.

**Figure 6:**
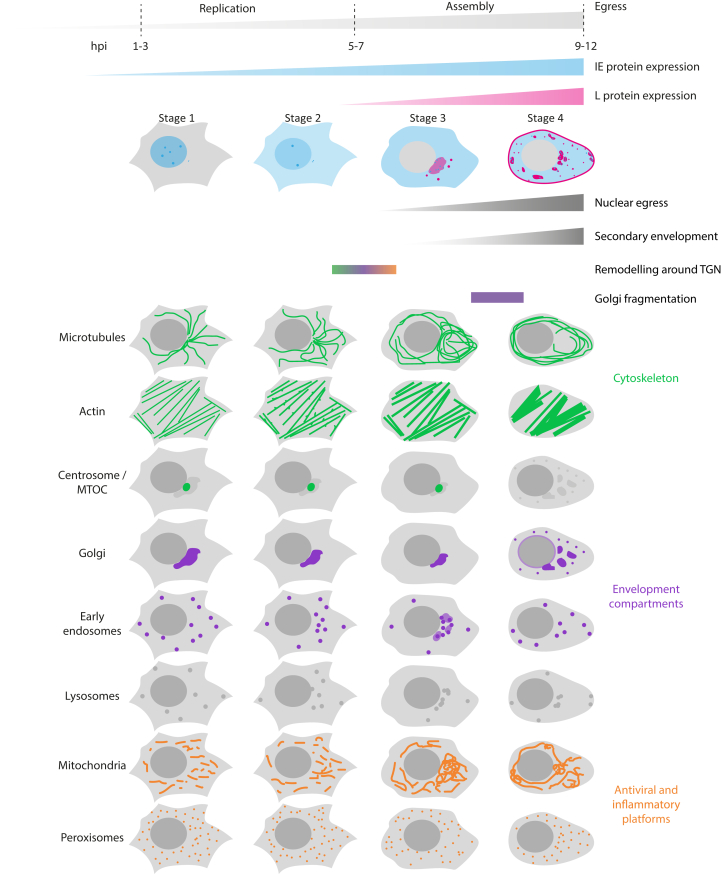
**Timeline of viral protein expression together with main organelle and cytoskeleton remodeling actions.** Below key events in the viral replication cycle and overview of spatiotemporal morphological changes determined by use of the timestamping virus.

Using our timestamping methodology, we provide the first detailed overview of virus-induced changes to a broad range of host cell structures and organelles with a temporal correlation to the HSV-1 replication cycle. We investigated three classes of cellular structures involved in the HSV-1 life cycle: the cytoskeleton (microtubules and actin), secretory and endocytic compartments (the Golgi apparatus and early endosomes), and known antiviral signaling platforms (mitochondria and peroxisomes). The observed changes are summarized in Figure 6.

We discovered two main subsets of concerted morphological changes. The first major remodeling takes place during the transition from stage 2 to stage 3 (see timeline in Fig. 6). During this transition, the late viral genes are expressed, which results in synthesis of the structural viral proteins necessary for virus assembly. Thus, we assume that the changes we observe are correlated to virus assembly. Central to the cellular rearrangement during the transition from stage 2 to stage 3 is the juxtanuclear region where the MTOC resides. Whereas microtubules seem to be pushed away from this part of the cell, early endosomes become clustered within this region alongside compaction of the Golgi complex, while mitochondria become intertwined with the juxtanuclear compartment enriched with viral glycoproteins. Previous studies speculated that mitochondria are accumulated within this region of the cell to supply energy for virus maturation, although no prominent activation of mitochondrial function could be observed, and a mechanism is still unknown (24). The relocation of early endosomes and potential fusion with the Golgi compartment that we observed fit well with the model of viral assembly where secondary envelopment occurs at membranes originating from the endocytic pathway and the trans-Golgi network.

The role of microtubules in herpesvirus transport and egress is well established. However, the clearance of microtubules away from the juxtanuclear region around the centrosome/MTOC is puzzling and might be related to inhibition of MTOC function. ICP0 has been reported to be responsible for the rearrangement of microtubules during HSV-1 infection (13) and could play a key role in this process. Our imaging data supports the role of ICP0 for microtubule reorganization during viral replication as we observed ICP0 colocalization with two markers of the MTOC/centrosome (SiR-tubulin and pericentrin) at stages 1 and 2, but then ICP0 only colocalized with SiR-tubulin and not pericentrin during stage 3, suggesting that HSV-1 modifies the organization of the peri-centriolar material of the MTOC/centrosome and that ICP0 associates more strongly with gamma-tubulin. Notably, the colocalization between ICP0 and the MTOC was more apparent in live than in fixed cells, which may explain why this has not been reported before.

Late in infection (stage 4), almost all cellular structures and organelles that we monitored underwent further profound rearrangement by this stage. These rearrangements were dominated by the fragmentation of the Golgi complex. This led to a redistribution of associated organelles and viral glycoproteins. Due to experimental differences in cell line and experimental conditions such as hpi and MOI, it can be difficult to compare our data with previously stated morphological changes observed during HSV-1 infection. However, in an attempt to compare the structural changes at stage 4 with literature reports, we selected studies that used high MOI and late times post infection, conditions for which cells in stage 4 should dominate the population. We found that our observations matched most descriptions of host cell rearrangements such as redistribution and bundling of microtubules (13–16 hpi, MOI 3–5) (25, 26), loss of centrosome/MTOC (13 hpi, MOI 5) (26), migration of mitochondria toward the juxtanuclear region (≥6 hpi, MOI 5) (24), and fragmentation of the Golgi apparatus (8–12 hpi, MOI 3–10) (25, 27). An exception were peroxisomes, where we did not observe previously described morphological changes, possibly because the published study used late times post infection (16 hpi, MOI 5) (28).

Using a fluorescent reporter virus with labels for capsid and viral envelope proteins, we found that appearance of capsids in the cytoplasm primarily starts during stage 3, but numbers are highly increased after fragmentation of the Golgi complex. It will be interesting to investigate if and how attachment of capsids to the glycoprotein-rich compact Golgi compartment leads to its fragmentation. Fragmentation generates a number of smaller compartments consisting of membranes that contain TGN and EE markers. It can be speculated that secondary envelopment, subsequent to nuclear egress, might occur mainly at fragmented, smaller membrane compartments, consistent with observations of virus wrapping sites in HFF cells by electron microscopy (15). It might be that small dispersed membrane compartments are advantageous for efficient secondary envelopment. The dispersed membrane compartments might be more accessible for capsids traveling through the cytoplasm after traversing the nuclear envelope as well as providing an overall increased membrane surface for attachment of tegument proteins.

We observe close association of mitochondria to the Golgi complex at the stage when gC accumulates at the membranes of Golgi and TGN (stage 3). With fragmentation of the Golgi and associated spread of gC membrane compartments, networks of elongated mitochondria that connect the clusters around gC-rich membranes become apparent. It has been shown that HSV-1 infection activates RIG-I (29), and in turn activation of the RLR pathway promotes elongation of the mitochondrial network, which modulates signaling downstream from MAVS (30). This indicates that the redirecting of the mitochondria to potential compartments of secondary envelopment is a host-cell response. It is not clear what causes the fragmentation of the Golgi complex and loss of the centrosome in HSV-1-infected cells, but both incidents have also been described during apoptosis (31, 32). Therefore, it seems plausible that during stage 4 morphological changes are caused by cytotoxic effects due to overloading the cell with late virus gene products and mature virions, rather than specific cell remodeling events by the virus to enhance replication. This correlates with the appearance of membrane blebbing and spikes that we observe during stage 4, which are also morphological characteristics of apoptosis (33).

Some of the changes we observed in our study are reminiscent of the changes occurring during infection with other herpesviruses, for example, human cytomegalovirus (HCMV), a betaherpesvirus. The kinetics of viral replication for HCMV and HSV-1 are very different (3–5 days compared with 8–12 h), but the reorganization of organelles exhibits striking similarities. During formation of HCMV assembly compartments (ACs) where virion tegumentation and envelopment take place, the host secretory and endocytic systems are dramatically reorganized, presumably at least in part by a shift in the balance between endocytic and exocytic recycling pathways (34). For example, it could be shown that vesicles positive for EE markers are concentrated at the center of ACs, further surrounded by membranes of the TGN and Golgi compartment (35, 36). Similarly, we detect for HSV-1 that EE accumulates at and partially fuses with membranes of potential compartments of secondary envelopment such as the TGN. Furthermore, HCMV exhibits control over host cell remodeling by enhancing the Golgi-based microtubule nucleation and suppresses centrosome activity so that the AC transforms into an MTOC (37). Likewise, it has been described for HSV-1 that formation of new MTOCs occurs at the TGN during HSV-1 infection (38), and consistently we find in our study that microtubules are remodeled around the Golgi compartment at transition from early to late stages in infection with ICP0 as a likely key player in the process. Another parallel between HSV-1 and HCMV is the temporal correlation of Golgi fragmentation with key events at late infection stages. Fragmentation of the Golgi membranes during HCMV infection is temporally linked to AC morphogenesis and virus production (36). For HSV-1, we observe that the bulk of capsid egress from the nucleus, which precedes tegumentation and secondary envelopment, coincides with Golgi fragmentation.

In summary, we have developed new technology that enables the direct, visual readout of the stage of viral replication on the single cell level. One advantage of our method lies in its applicability in both live and fixed cells, and this methodology could prove useful for the study of other important viral pathogens where viral genes with different kinetic and spatial expression patterns can be effectively tagged with fluorescent reporters. In addition to linking morphological remodeling of the host cell to phases during the viral replication cycle, the tools we have developed can also be used to investigate the temporal correlation between HSV-1 replication and changes in, for example, cellular dynamics, mechanical properties, and metabolic function. In the future, our approach can also be used to identify viral genes that are involved in the remodeling of the host cell through the introduction of mutations into the timestamping virus. This will therefore pave the way for gaining substantial new understanding on virus–host interactions based on high-resolution single-cell imaging.

## Experimental Procedures

### Antibodies and reagents

Primary antibodies anti-EEA1 (ab70521), anti-58K Golgi (ab27043), antipericentrin (ab28144), and anticatalase (ab110292) were purchased from Abcam, anti-TGN46-8 (SAB4200355) from Sigma and anti-Tom20 (sc-17764) from Santa Cruz Biotechnology. All primary antibodies were monoclonal mouse antibodies. Polyclonal secondary antimouse antibodies conjugated to Atto647N (50185, Sigma Aldrich) as well as isotype-specific secondary antimouse antibodies conjugated to Atto647N (610-156-041, Rockland Immunochemicals) and Alexa Fluor 568 (A-21124, Thermo Fisher Scientific) were used for detection. The nanoboosters—single-domain alpaca antibody fragments covalently coupled to fluorescent dyes—GFP-Booster Atto488 and RFP-Booster Atto594 (Chromotek) were used to boost eYFP and mCherry fluorescence, respectively. Live cell stains SiR-tubulin, SiR-actin, and SiR-lysosome were from Spirochrome and MitoTracker Deep Red FM was from Thermo Fisher Scientific.

### Cell lines and plasmids

Human foreskin fibroblast (HFF-hTERT) cells (39) were cultured with Dulbecco's Modified Eagle's Medium (DMEM, high glucose) supplemented with 10% fetal bovine serum, 1% GlutaMAX, 100 U/ml penicillin, and 100 μg/ml streptomycin at 37 °C and 5% CO_2_. Plasmids mIFP12-Rab4a-7 (#56261) and mIFP-Golgi-7 (#56221) were purchased from Addgene.

### Cloning and generation of mIFP-expressing stable cell lines

The mIFP-tagged constructs were amplified by PCR using primers containing flanking Gateway attB sequences and cloned into the Gateway donor vector pDONR223. Constructs were subsequently cloned into lentiviral destination vector pHAGE-pSFFV using the Gateway system (Thermo Scientific). Lentiviral particles were generated by transfection of HEK293T cells with the pHAGE-pSFFV vectors plus four helper plasmids (VSVG, TAT1B, MGPM2, CMV-Rev1B), using TransIT-293 transfection reagent (Mirus) according to the manufacturer's recommendations. Lentiviral supernatants were harvested 48 h after transfection, cell debris was removed with a 0.22 μm filter, and HFF-hTERT cells were transduced for 48 h and then subjected to puromycin selection to generate stable cell populations expressing mIFP-tagged markers.

### Recombinant viruses

Fluorescently labeled, recombinant eYFP-ICP0/gC-mCherry (timestamp reporter) and eYFP-VP26/gM-mCherry viruses were constructed using the bacterial artificial chromosome (BAC)-cloned KOS strain of HSV-1 (40) and the two-step Red recombination technique (41) using the primers shown in Table S1. One or two rounds of two-step Red recombination with wild-type KOS BAC were conducted to firstly insert the eYFP(A206K) coding sequence in frame at the 5’ end of the UL35 gene (VP26) or at the 5’ end of both copies of the RL2 gene (ICP0) respectively. Insertion of the mCherry coding sequence immediately before the stop codon of the UL10 gene (gM) or the UL44 gene (gC) was then conducted with a further round of two-step Red recombination with the appropriate eYFP(A206K) containing BAC constructs. Infectious virus was reconstituted by transfection of Vero cells with BAC DNA together with a Cre recombinase expression plasmid to excise the BAC-cassette. All virus stocks were grown in Vero cells and infectious titres determined by plaque assay on HFF-hTERT and Vero cells.

### Growth curves and plaque assays

Single-step growth curves were conducted using HFF-hTERT cells infected in complete media with HSV-1 KOS WT or HSV-1 eYFP-ICP0/gC-mCherry at MOI of 10. After adsorption for 1 h at 37 °C, cells were incubated with acid wash (40 mM citric acid, 135 mM NaCl, 10 mM KCl; pH 3.0) for 1 min and washed 3x with PBS before cell culture media was added back. The time of acid wash was deemed 0 hpi. At various times postinfection, cells were harvested by freezing the plate at −70 °C. After freezing the last time point, samples were freeze-thawed together two subsequent times and scraped before they were titred. Titrations were performed on Vero monolayers. Cells were inoculated with serial dilutions of the samples for 1 h, after which DMEM containing 0.3% high viscosity carboxymethyl cellulose, 0.3% low viscosity carboxymethyl cellulose, 2% (v/v) FBS, 2 mM L-glutamine, 100 U/ml penicillin, and 100 μg/ml streptomycin was overlaid. After 3 days, cells were fixed in 3.75% (v/v) formaldehyde in PBS for 30 min and stained with 0.1% toluidine blue.

### Infection assay

For infection assays, HFF cells were plated in 8-well LabTek coverglass chambers at 25,000 cells per well or onto 13 mm diameter round coverslips in 4-well plates at 60,000 cells per well 12 h prior to infection. The next day, cells were infected with recombinant virus at 3 PFU per cell if not indicated otherwise. After 1 h of incubation at 37 °C and 5% CO_2_, medium was exchanged. Depending on the experiment, samples were transferred onto the respective microscope equipped with appropriate environmental control for live cell imaging after 3hpi or fixed, permeabilized, and labeled with antibodies at different hpi as indicated and according to procedures described below.

### Live cell staining

For live cell imaging, samples were stained with either the silicon rhodamine (SiR) dyes SiR-tubulin, SiR-actin or SiR-lysosome or MitoTracker Deep Red FM half an hour prior to imaging. The SiR dyes are far-red, fluorogenic, cell-permeable, and highly specific live cell probes compatible with eYFP and mCherry. For staining of microtubules, actin, or lysosomes, cells were incubated with a solution of 1 µM respective SiR dye and 10 µM verapamil in DMEM with supplements. Verapamil is an ion channel inhibitor and used to reduce activity of efflux pumps, leading to an enhanced signal intensity of SiR dyes. SiR dyes were left on the sample for imaging. For staining of mitochondria, cells were incubated with a solution of 20 nM MitoTracker Deep Red FM in DMEM with supplements. After half an hour incubation, cells were washed two times with prewarmed cell culture medium and immediately transferred to the microscope.

### Immunostaining

Cells were immunostained using a standard immunofluorescence protocol. For fixation, cells were incubated in PBS with 4% methanol-free formaldehyde (28906, Thermo Fisher Scientific, Waltham, USA) for 10 min at room temperature. After permeabilization in PBS with 0.2% (v/v) TritonX-100 for 5 min and blocking with 10% goat serum in PBS for 45 min, samples were incubated at room temperature for 1 h with primary antibody (1:200 dilution) in PBS solution containing 3% bovine serum albumin (BSA). After washing three times with PBS, cells were incubated with Atto647N-labeled secondary antibody (1:400 dilution in PBS with 3% BSA) for 30 min at room temperature in the dark. After washing three times with PBS, cells were postfixed with formaldehyde as described before.

Samples that were expanded as described below were first fixed with 4% methanol-free formaldehyde (28906, Thermo Fisher Scientific, Waltham, USA) and 0.1% glutaraldehyde (G7651, Sigma Aldrich, Darmstadt, Germany) in PBS for 10 min and permeabilized by incubation with a Tween 20 solution (0.5% v/v in PBS) for 15 min at room temperature. After one wash in PBS, cells were incubated with blocking buffer (10% goat serum and 0.05% Tween 20 in PBS) for 30 min at room temperature. Samples were then incubated with the primary antibody (1:200 dilution) in blocking buffer at 4 °C overnight, washed three times with PBS over 15 min, and incubated with the conjugated secondary antibody (1:400 dilution) in blocking buffer for 1 h at room temperature in the dark. After washing three times with PBS over 15 min, a nanobooster was used to enhance fluorescence of mCherry-tags with a synthetic dye, which is brighter and more resistant to the expansion protocol than the fluorescent protein tags. Cells were incubated with nanoboosters for eYFP and mCherry diluted 1:200 in PBS containing 4% BSA and 0.05% Tween 20 for 1 h at room temperature in the dark. Samples were washed three times with PBS over 15 min and expanded the next day.

### Gelation, digestion, and expansion of HSV-1 infected cells

Infected, fixed, and immunostained cells were expanded following the expansion protocol described by Chozinski *et al.* (42), which is compatible with conventional synthetic dyes and autofluorescent protein tags. Fixed cell samples on 13 mm diameter round coverglass were incubated in monomer solution (2 M NaCl, 2.5% w/w acrylamide, 0.15% w/w N,N'-methylenebisacrylamide, 8.625% w/w sodium acrylate in PBS) for ∼1 min at room temperature prior to gelation. Concentrated stocks of ammonium persulfate (APS) and tetramethylethylenediamine (TEMED) at 10% (w/w) in water were diluted in monomer solution to concentrations of 0.2% (w/w) for gelation, with the initiator (APS) added last. The gelation solution (70 μl) was placed in a 1 mm deep, 1 cm diameter Teflon well, and the coverglass was placed on top of the solution with cells face down. After 30 min at room temperature, gelation was complete. The coverglass and gel were removed with tweezers and placed in digestion buffer (1× TAE buffer, 0.5% Triton X-100, 0.8 M guanidine HCl) containing 8 units/ml proteinase K (17916, Thermo Fisher Scientific, Waltham, USA) freshly added. Gels were digested at 37 °C for a maximum of 30 min. The gels were removed from digestion buffer and placed in 50 ml DI water to expand. Water was exchanged every 30 min until expansion was complete (typically 3–4 exchanges).

### Widefield microscopy

Time-lapse imaging of live HSV-1 infected cells was carried out at a custom-built widefield microscope. Frame (IX83, Olympus, Tokyo, Japan), stage (Prior, Fulbourn, UK), Z drift compensator (IX3-ZDC2, Olympus, Tokyo, Japan), plasma light source (HPLS343, Thorlabs, Newton, USA), and camera (Clara interline CCD camera, Andor, Belfast, UK) were controlled by Micro-manager (43). Respective filter cubes for YFP (excitation 500 nm, dichroic mirror 515 nm, emission 535 nm), mCherry (excitation 560 nm, dichroic mirror 585 nm, emission 630 nm), Atto647N (excitation 635 nm, dichroic mirror 660 nm, emission 680 nm), and DAPI (excitation 350 nm, dichroic mirror 353 nm, emission 460 nm) were used. Images were acquired with an Olympus PlanApoU 60x/1.42 oil objective lens at 20 to 25 random positions for each sample. For 12 h, images were recorded every 20 min starting at 3.5 hpi for ICP0-eYFP/gC-mCherry and at 8 hpi for VP26-eYFP/gM-mCherry HSV-1. During the experiment, cells were heated at 37 °C and 5% CO_2_ *via* a stage top incubator and temperature, gas, and humidity controllers (Okolab, Pozzuoli, Italy). Volumes of fixed cells immunostained for TOM20 were acquired as z-stacks with a step size of 0.3 μm over a range of 9 μm. Data were deconvolved and maximum intensity projected as described below.

### Structured illumination microscopy (SIM)

Structured illumination images were collected on a custom-built SIM setup, modified from one that has been described before in detail (44). A 60×/1.2 NA water immersion lens (UPLSAPO60XW, Olympus, Tokyo, Japan) was used to focus the structured illumination pattern onto the sample and captured the samples' fluorescence emission light, which was detected with a sCMOS camera (C11440, Hamamatsu, Hamamatsu-City, Japan). Laser excitation wavelengths used were 488 nm (iBEAM-SMART-488, Toptica, Graefelfing, Germany), 561 nm (OBIS561, Coherent, Santa Clara, USA), and 640 nm (MLD640, Cobolt, Solna, Sweden). Respective emission filters were BA510-550 (Olympus, Tokyo, Japan), BrightLineFF01-600/37, and BrightLineFF01-676/29 (Semrock, NewYork, USA). Imaging was done in fixed or live cells, as indicated. During the live cell experiment, cells were heated at 37 °C and supplied with 5% CO_2_ *via* a stage top incubator and temperature, gas, and humidity controllers (Okolab, Pozzuoli, Italy). Images were acquired using HCImage (Hamamatsu, Hamamatsu-City, Japan) and a custom-built control software based on Labview (National Instruments, Newbury, UK). Nine raw images were collected at each plane and each color. Raw SIM images were reconstructed with FairSIM (45) using a customized Fiji macro including correction for the lateral shift between colors, which was measured using 100 nm beads (TetraSpeck Microspheres, Thermo Fisher Scientific, Waltham, USA). Out-of-focus light was suppressed by intermixing separated bands as suggested by Shaw *et al.* (46).

### Light sheet microscopy

Expanded samples were imaged at a custom-built inverted selective plane illumination microscope (iSPIM). Parts were purchased from Applied Scientific Instrumentation (ASI, Eugene, USA) including controller (TG8_BASIC), scanner unit (MM-SCAN_1.2), right-angle objective mounting (SPIM-K2), stage (MS-2K-SPIM) with motorized Z support (100 mm travel range, Dual-LS-100-FTP), and filter wheel (FW-1000-8). All components were controlled by Micro-Manager by means of the diSPIM plugin. The setup was equipped with a 0.3 NA excitation objective (Nikon 10x, 3.5 mm working distance) and a higher, 0.9 NA detection objective (Zeiss, W Plan-Apochromat 63 × 2.4 mm working distance) to increase spatial resolution and fluorescence signal collection. Lasers (OBIS488-150 LS, OBIS561-150 LS and OBIS647-120 LX, Coherent, Santa Clara, USA) were fiber-coupled into the scanner unit. An sCMOS camera (ORCA-Flash 4.0, Hamamatsu, Hamamatsu-City, Japan) was used to capture fluorescence. Respective emission filters were BrightLineFF01-540/50, BrightLineFF01-624/40 and BrightLineFF0-680/42 (Semrock, NewYork, USA). Gels containing expanded samples were cut into small strips and mounted onto 24 × 50 mm rectangular coverslips with expanded cells facing upward using Loctite super glue (Henkel, Duesseldorf, Germany). The sample was then placed into an imaging chamber (ASI, I-3078-2450), which was filled with water. We recorded 150 planes per volume and color channel, spacing planes every 0.5 μm. Raw data were deskewed using a custom Fiji plugin.

### Deconvolution

Z-stacks taken at the widefield microscope and deskewed light sheet microscopy data were deconvolved using a custom GPU-accelerated chunked deconvolution routine written in MATLAB. In total, 250 iterations of the unregularized Richardson–Lucy algorithm were used on an initial estimate set to the mean value of the raw data, following a background subtraction to correct for camera offset. Deconvolved data were maximum intensity projected in Fiji, using color to indicate depth as indicated.

### Cell counting and assignment of stage of infection

Cells were seeded onto 13 mm diameter round coverslips as described above and infected the next day with eYFP-ICP0/gC-mCherry virus at MOI 0.3, 3, and 30, respectively. Samples were fixed every hour after infection, starting at 3 hpi until 8 hpi. In order to increase fluorescent signal of eYFP- and mCherry-tags of the reporter virus, cells were immunostained with nanoboosters. Samples were then mounted in mounting medium containing DAPI (VECTASHIELD HardSet Mounting Medium, Vectorlabs) according to standard manufacturer's protocol. For each condition, 20 to 25 FOVs were acquired at the widefield microscope as described above with 5 to 20 cells per FOV. Nuclei were counted to yield the total number of cells and each individual cell was then classified as noninfected or assigned a stage of infection according to the classification scheme. For each condition, between 135 and 300 cells were counted.

### Measurement of organelle area and particle numbers

The area occupied by the Golgi complex and peroxisomes was determined from reconstructed SIM images and for gM compartments from widefield images using Fiji. For the Golgi complex, the outline of the compartment was traced manually, whereas for peroxisomes binary images were created by use of Otsu thresholding. For gM compartments, the background was first subtracted using a rolling ball radius of 20 pixel before Otsu thresholding. The area of the compartments was then determined using the “Analyze Particles” function. Capsids were localized and counted with the TrackMate plugin for Fiji.

### Colocalization analysis

In order to calculate the overlap between gC and TGN compartments, images for both channels were first segmented using Fiji. Masks were created by using the adjust brightness/contrast function. An overlap mask was created by using the AND operation in the image calculator function. Areas were measured and the fraction of overlap area to compartment area (gC or TGN, respectively) was calculated.

### Density maps

SIM images of vesicular structures were Gaussian filtered (10 pixel) in order to smooth edges of fluorescent structures using Fiji. For highlighting regions with a high density of vesicular structures, the lookup table ICA was chosen.

### Skeleton analysis

Properties of the mitochondria networks were analyzed using Fiji. Widefield z-stacks of mitochondria were first filtered by subtracting the background using a five-pixel rolling ball radius. Stacks of binary mask images were created by Otsu intensity thresholding. Thresholding artifacts were removed by using the “Despeckle” function. The images were then skeletonized and the 3D skeletons analyzed using the available Fiji functions. Data was represented in graphs using GraphPad Prism 8 (GraphPad Software, San Diego, USA).

## Data availability

All data are contained within the article.

## Conflict of interest

The authors declare that they have no conflicts of interest with the contents of this article.
