# Supplementary material for: A fluorescent reporter system enables spatiotemporal analysis of host cell modification during herpes simplex virus-1 replication
Source: J Biol Chem. 2021 Jan 7;296:100236. doi: 10.1074/jbc.RA120.016571 (PMC7948757; doi:10.1074/jbc.RA120.016571)
Supplement: Supplementary Figures and Table [file mmc3.pdf]

Supporting Information

Spatiotemporal analysis of host cell modification during herpes simplex virus-1 replication

Katharina M. Scherer[1], James D. Manton[1,2], Timothy K. Soh[3,4], Luca Mascheroni[1], Vivienne Connor[3], Colin M. Crump[3,*], Clemens F. Kaminski[1,*]

[1]Department of Chemical Engineering & Biotechnology, University of Cambridge, CB3 0AS, UK
[2]MRC Laboratory of Molecular Biology, Francis Crick Avenue, Cambridge, CB2 0QH, UK
[3]Department of Pathology, University of Cambridge, CB2 1QP, UK
[4]Present address: Heinrich-Pette-Institute, Martinistrasse 52, 20251 Hamburg, Germany

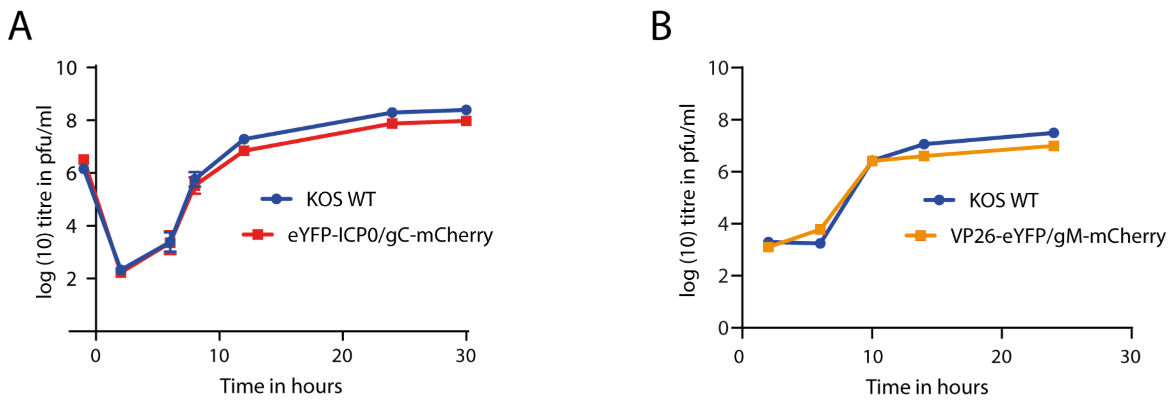

Supporting Figure 1: High MOI growth curves. Cells were infected at a MOI of 10 for 1 h at 37˚C, treated with acid wash to neutralise residual infectivity and then incubated in culture media for the specified times. Total virus production at each time point was determined by plaque assay on Vero cell monolayers. (**A**) HFF-hTERT cells were infected with wildtype virus and the eYFP-ICP0/gC-mCherry mutant. Three biological replicates were used for each condition. Data represents mean infectious titre and error bars show the standard deviation. (**B**) Vero cells were infected with wildtype virus and the VP26-eYFP/gM-mCherry mutant. Two biological replicates were used for each condition. Data represents mean infectious titre and error bars show the standard deviation.

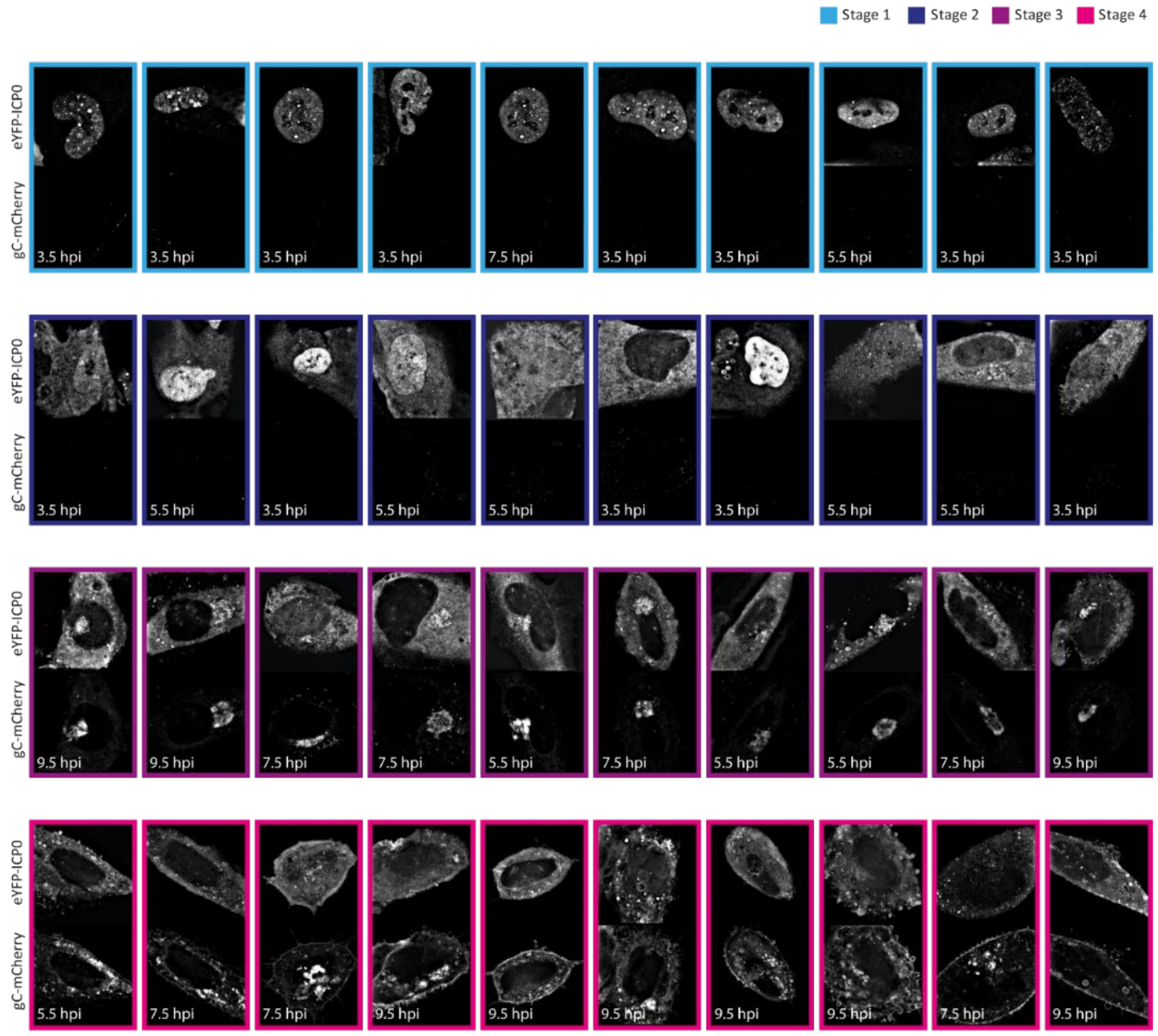

Supporting Figure 2: Variation in the stage of infection at the individual cell level at different hours post infection. HFF cells were infected with eYFP-ICP0/gC-mCherry HSV-1 and fixed at 3.5, 5.5, 7.5 and 9.5 hpi. Using SIM, visual features determined by eYFP-ICP0 and gC-mCherry appearance and distribution were applied to classify each individual cell. Despite different cell shapes, features are mostly uniform for each stage of infection. Exception is stage 2 which is characterized by the transition of ICP0 from the nucleus into the cytoplasm. Individual cells captured during the transition can appear with ICP0 being present still predominantly in the nucleus, with equal amounts in the nucleus and the cytoplasm, or predominantly in the cytoplasm in different cells.

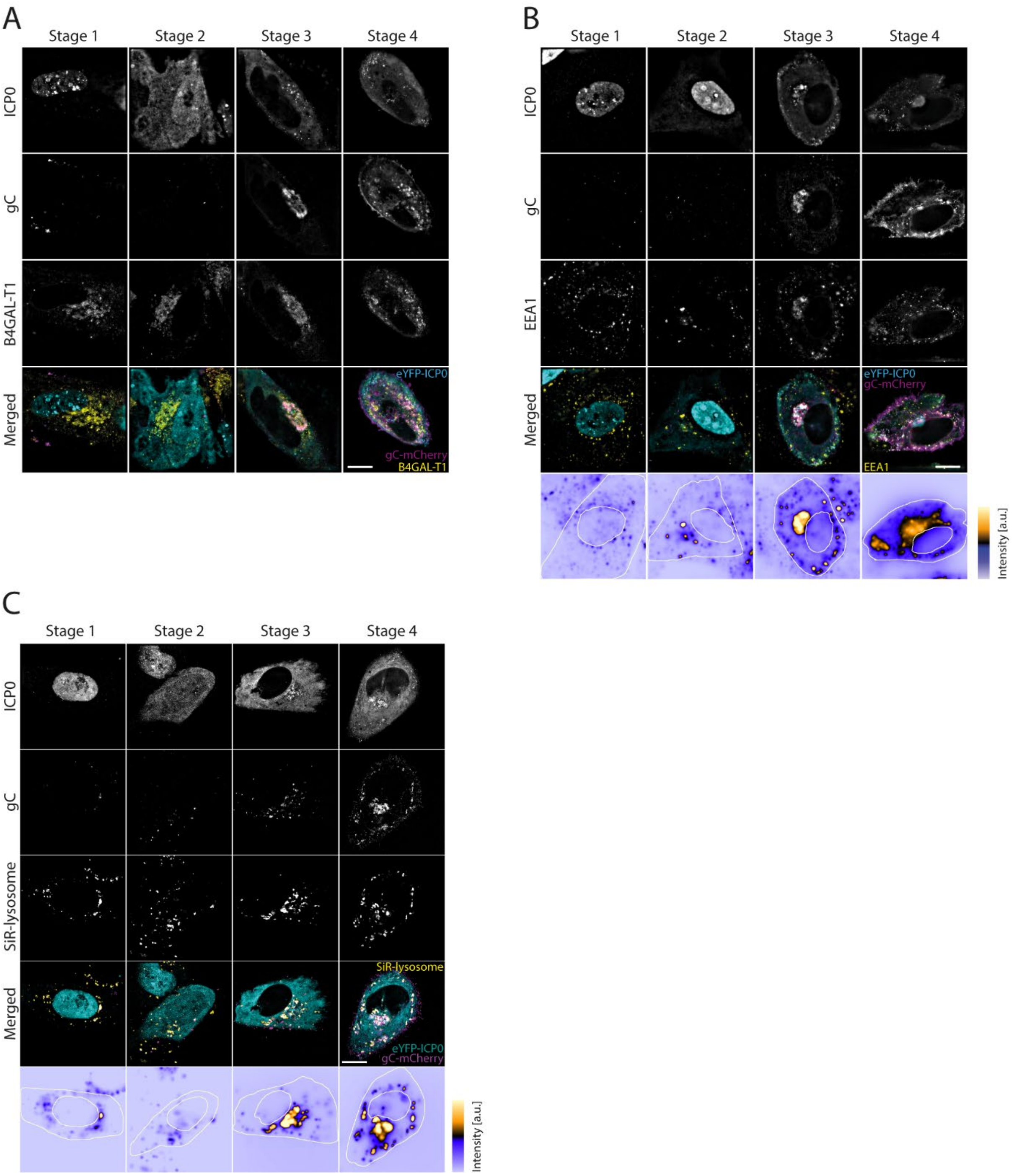

Supporting Figure 3: **(A)** Morphology changes and fragmentation of the Golgi complex during HSV-1 replication. HFF cells stably expressing mIFP-B4GAL-T1 were infected with eYFP-ICP0/gC-mCherry virus, fixed at 3.5, 5.5, 7.5 and 9.5 hpi and imaged using SIM. **(B)** Spatial distribution of early endosomes. HFF cells were infected with eYFP-ICP0/gC-mCherry HSV-1, and fixed at 3.5, 5.5, 7.5 and 9.5 hpi. They were stained for early endosomes with an antibody against EEA1 by use of a standard immunofluorescence protocol and imaged using SIM. Early endosomes accumulate at the juxtanuclear region during stage 2 and 3, and re-distribute in the cytoplasm during stage 4 due to fragmentation of juxtanuclear compartment. **(C)** Lysosome distribution during all stages of infection. HFF cells were infected with eYFP-ICP0/gC-mCherry HSV-1, stained with SiR-lysosome and imaged live at 3.5, 5.5, 7.5 and 9.5 hpi using SIM. Lysosomes are attracted to compartments rich in gC. Scale bars 10 µm.

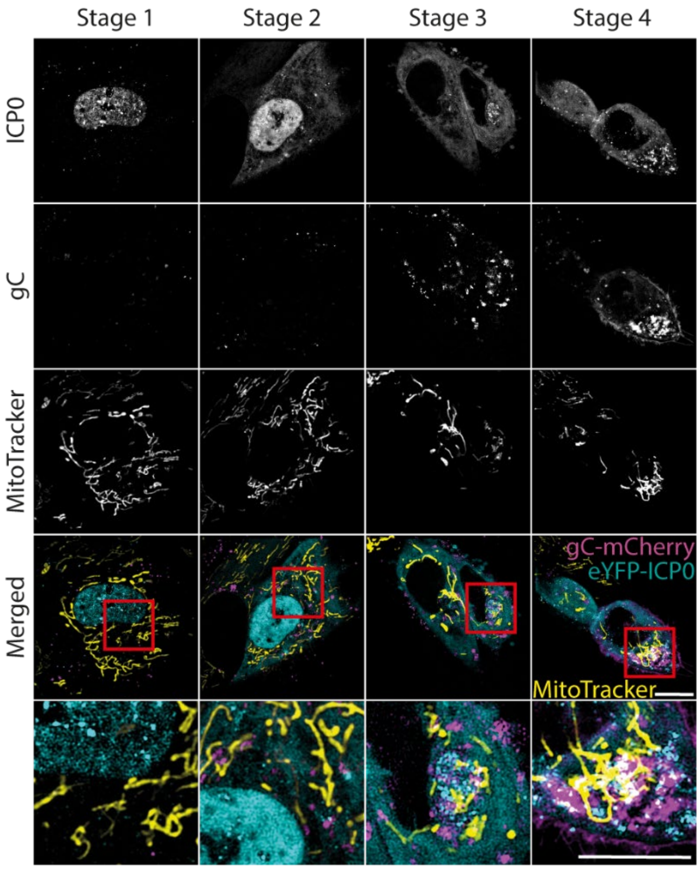

Supporting Figure 4: Mitochondria re-location towards gC-rich compartments. HFF cells were infected with eYFP-ICP0/gC-mCherry HSV-1, stained with MitoTrackerDeepRed and imaged live at 3.5, 5.5, 7.5 and 9.5 hpi using SIM. Scale bar 10 µm.

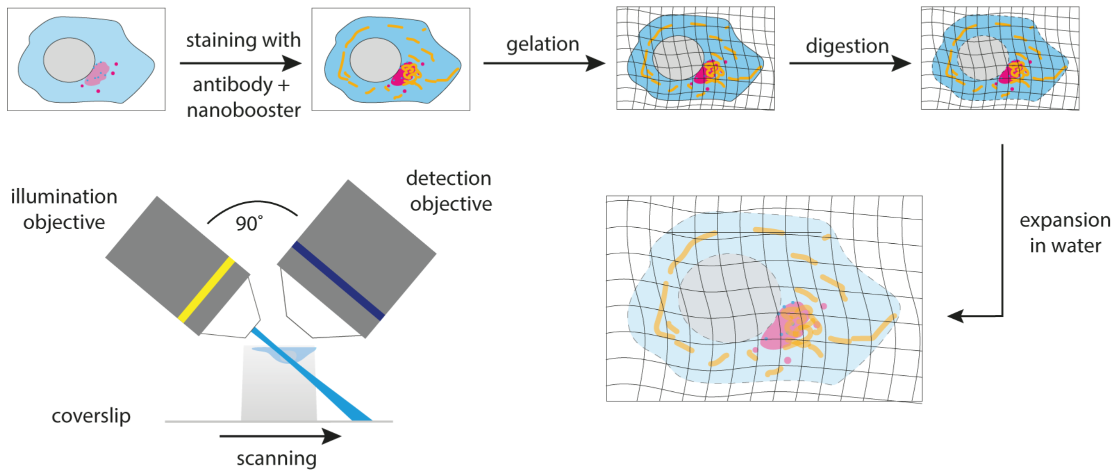

Supporting Figure 5: Schematic illustration of expansion microscopy and light sheet microscopy. Infected cells were fixed, and structures of interest were stained using a standard immunofluorescence protocol. A nanobooster specific for mCherry was used to enhance the fluorescence signal from the viral protein gC. Expanded samples were cut into thin strips and mounted onto coverslips with superglue for imaging on a custom-built, inverted light sheet microscope.

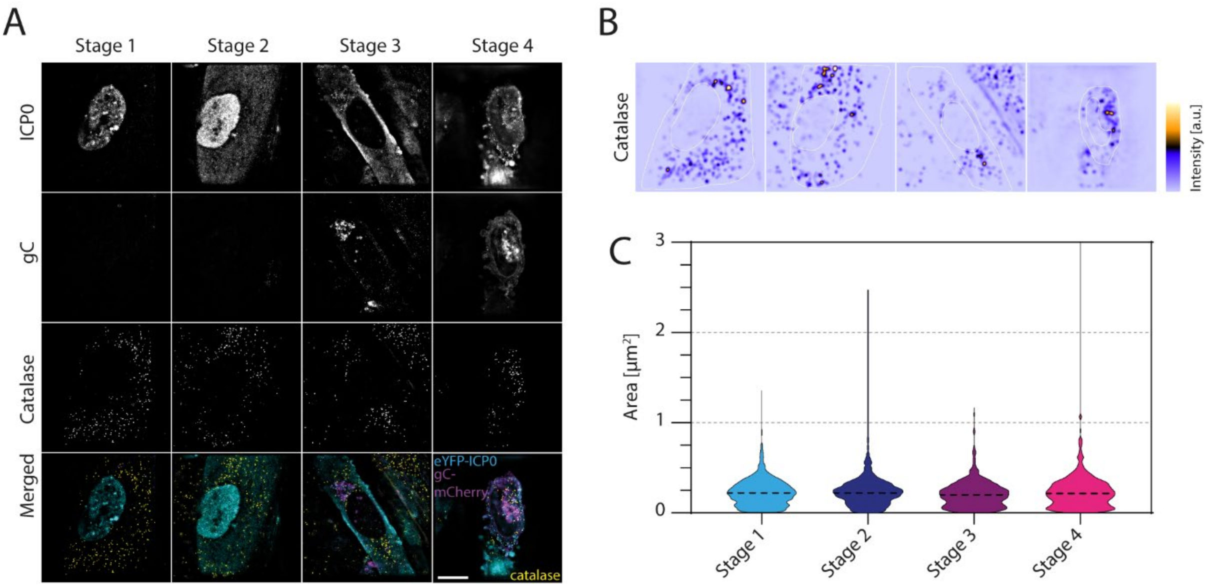

Supporting Figure 6: **(A)** HFF cells were infected with eYFP-ICP0/gC-mCherry HSV-1, fixed at 3.5, 5.5, 7.5 and 9.5 hpi. They were stained for peroxisomes with an antibody against catalase by use of a standard immunofluorescence protocol and imaged using SIM. **(B)** No change of spatial distribution is obvious. **(C)** Distribution of peroxisome size. The horizontal line in the violin plot indicates the median of the distribution (stage 1: 1623 peroxisomes in 11 cells, stage 2: 948 peroxisomes in 8 cells, stage 3: 401 peroxisomes in 4 cells, stage 4: 809 peroxisomes in 12 cells). Scale bars 10 µm.

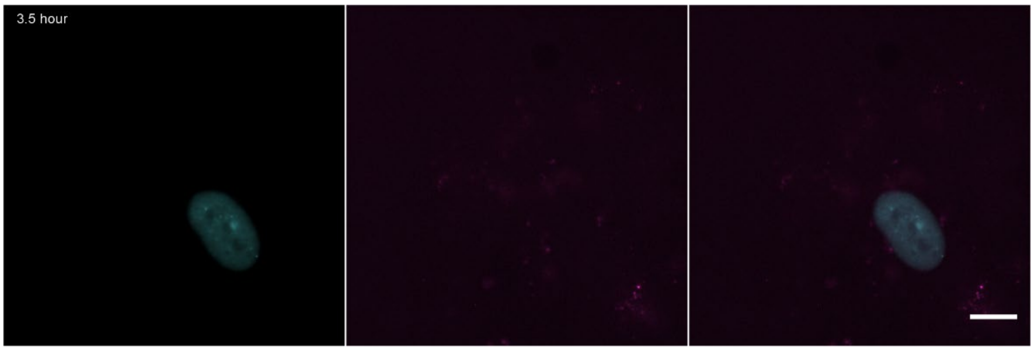

Supporting Video 1: Time-lapse imaging of HFF cells infected with eYFP-ICP0/gC-mCherry HSV-1. Imaging was started 3.5 hpi, and images were recorded for 12 hours every 20 min. The transition of eYFP-ICP0 from the nucleus into the cytoplasm as well as the expression of gC-mCherry late in infection can be clearly observed confirming the functionality of our reporter virus.

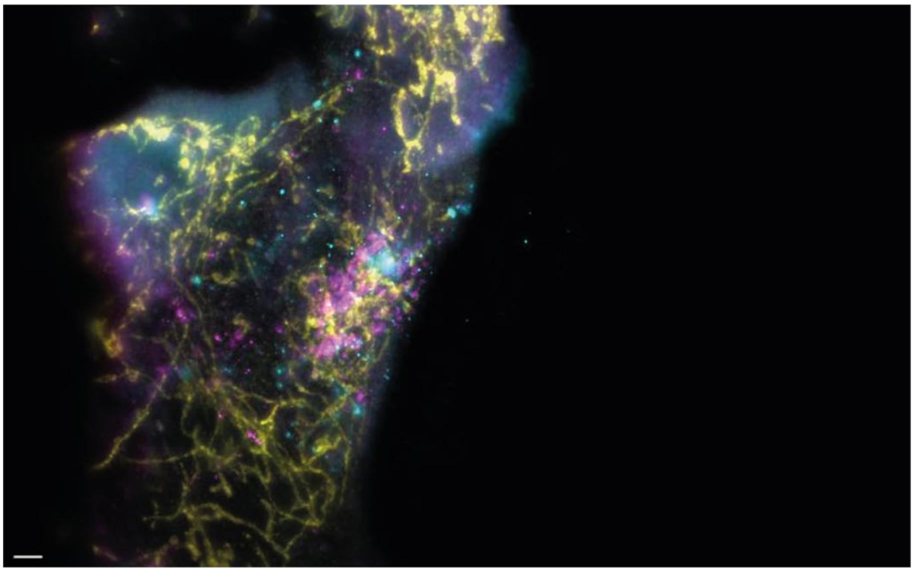

Supporting Video 2: Interlacing of mitochondria with viral assembly compartment at stage 3 of infection. Infected cells were fixed 7.5 hpi, and immunostained for TOM20. Fluorescence of eYFP and mCherry was enhanced by use of nanoboosters. Samples were expanded 4x and imaged at a custom-built light sheet microscope. The video corresponds to Figure 4b in the main text.

Supplementary Table 1: Primers for cloning the recombinant, fluorescent viruses.

Name	Sequence (5′–3′)	Details
COL585	CCCCCAGGGACCCTCCGTCAGCGACC<br>CTCCAGCCGCATACGACCCCCATGGT<br>GAGCAAGGGCGAGGAG	Forward primer for inserting in frame eYFP(A206K) at the N-terminus of ICP0
COL586	CGCTGGGGGCGGCCCTCAGGCCGGCG<br>GGTACTCGCTCCGGGGCGGGGCTCCTT<br>GTACAGCTCGTCCATG	Reverse primer for inserting in frame eYFP(A206K) at the N-terminus of ICP0
COL650	CAATCGTGTACGTCGTCCGCACATCAC<br>AGTCGCGGCAGCGTCATCGGCGGGTG<br>AGCAAGGGCGAGGAG	Forward primer for inserting in frame mCherry at the C-terminus of gC
COL651	GGGACCAAACTATATAGATATTAAAA<br>AGGTAACGGGGGGATCTCGCGTTACT<br>TGTACAGCTCGTCCATG	Reverse primer for inserting in frame mCherry at the C-terminus of gC
SS07	ACAGCCCTCCCGTCCGACACCCCCATA<br>TCGCTTCCCGACCTCCGGTCCCGATGG<br>TGAGCAAGGGCGAGGAG	Forward primer for inserting in frame eYFP(A206K) in place of amino acids 1-4 at the N-terminus of VP26
SS08	CCAAGCGCCCGGACGCTATCGGTGGT<br>AACGGTGCTGGGGCGGTGAAATTG<br>CTTGTACAGCTCGTCCATGCC	Reverse primer for inserting in frame eYFP(A206K) in place of amino acids 1-4 at the N-terminus of VP26
SS11	ACGACCCCGAGCCCGCCGAGGACCCC<br>GTGTACAGCACCGTCCGCCGTTGGGT<br>GAGCAAGGGCGAGGAG	Forward primer for inserting in frame mCherry at the C-terminus of gM
SS12	CACCACGGTCGGGTTAAACACAAACG<br>GTTTATTAAAACGGAACCAAACAGTT<br>ACTTGTACAGCTCGTCCATG	Reverse primer for inserting in frame mCherry at the C-terminus of gM
